# Cations Form Sequence Selective Motifs within DNA Grooves via a Combination of Cation-Pi and Ion-Dipole/Hydrogen Bond Interactions

**DOI:** 10.1371/journal.pone.0071420

**Published:** 2013-08-06

**Authors:** Mikaela Stewart, Tori Dunlap, Elizabeth Dourlain, Bryce Grant, Lori McFail-Isom

**Affiliations:** Department of Chemistry, University of Central Arkansas, Conway, Arkansas, United States of America; Florida International University, United States of America

## Abstract

The fine conformational subtleties of DNA structure modulate many fundamental cellular processes including gene activation/repression, cellular division, and DNA repair. Most of these cellular processes rely on the conformational heterogeneity of specific DNA sequences. Factors including those structural characteristics inherent in the particular base sequence as well as those induced through interaction with solvent components combine to produce fine DNA structural variation including helical flexibility and conformation. Cation-pi interactions between solvent cations or their first hydration shell waters and the faces of DNA bases form sequence selectively and contribute to DNA structural heterogeneity. In this paper, we detect and characterize the binding patterns found in cation-pi interactions between solvent cations and DNA bases in a set of high resolution x-ray crystal structures. Specifically, we found that monovalent cations (Tl^+^) and the polarized first hydration shell waters of divalent cations (Mg^2+^, Ca^2+^) form cation-pi interactions with DNA bases stabilizing unstacked conformations. When these cation-pi interactions are combined with electrostatic interactions a pattern of specific binding motifs is formed within the grooves.

## Introduction

Complex arrays of electronic and steric factors combine to influence and modulate the fine, dynamic subtleties of DNA structure. These subtleties in turn provide a fundamental component of sequence dependent protein binding, gene activation/repression, and DNA repair. DNA sequence is communicated in these processes through both the pattern of hydrogen bond donors and acceptors lining the groove floors as well as the conformational heterogeneity including flexibility and curvature imparted indirectly by the sequence.

Cation-pi interactions between solvent cations or their first hydration shell waters (FSWs) and the faces of DNA bases have been observed in DNA structures. Solvent cations form electrostatic interactions with both the negatively charged DNA phosphate backbone and the hydrogen bond acceptors lining the groove floor. In this way, cations positioned within the grooves are positioned by the DNA sequence and in turn, modulate DNA conformational heterogeneity including groove narrowing [1] and base unstacking [2]. Previously, we reported two instances of base unstacking stabilized through cation-pi interactions between the base aromatic faces and Mg^2+^ FSWs [3]. However, with only two cation-pi interactions available for inspection, speculation regarding sequence selectivity was difficult since the varied and detailed observations required for general pattern detection were lacking. Cation-pi interactions between divalent cation FSW and the aromatic face of DNA bases were subsequently reported by Howerton, et al who observed Tl^+^ (a K^+^ mimic) ions docked cleanly on the faces of terminal cytosines [4].

Cation-pi interactions originally characterized by Ma and Dougherty [5], [6] have also been established as fundamental interactions involved in many levels of protein structure [7], [8], [9], [10]. In proteins, these interactions occur most frequently between positively charged arginine side chains and aromatic side chains such as tryptophan, phenylalanine, and tyrosine (reviewed in [10], [11]). Cation-pi interactions have also been established as a stabilizing interaction between aromatic side chains and hydrogen bond donors [12] or anionic side chains [13], [14]. Cation-pi interactions are thought to contribute to a disparate and eclectic range of systems including the binding of a wide array of neurotransmitters to their receptors [15], [16], [17], [18], induction of kinking in a hinge region of RNA polymerase [19], apoA1/HDL structure and function reviewed in [20] and the detection of alkylated bases [21] and in DNA repair [22].

However, when cation-pi interactions were first proposed some computational analyses discounted the stability and contribution of cation-pi interactions between cations/FSWs and DNA bases [23], [24], [25]. Most of the objections arose from weak interaction energies and long interaction distances predicted by the theoretical methods and these objections have been subsequently explained or refuted [26], [27]. Computational methods also support the importance of cation-pi complexes involving DNA bases and monovalent cations such Na^+^ [28].

In this article, missing details are revealed through the analysis of a variety of cation-pi interactions between solvent cations/FSWs and DNA bases. These cation-pi interactions were detected through the application of strict criteria to high resolution DNA crystal structures. Specifically, we find a variety of cations, including monovalent Tl^+^ and divalent Mg^2+^ and Ca^2+^, interact with the faces of DNA bases either directly (as with Tl^+^) or via the polarized first shell waters (FSWs) of divalent cations. Furthermore, these cation-pi interactions stabilize unstacked DNA bases, form sequence selectively in both grooves, and when combined with favorable electrostatic interactions with adjacent base edges, predictably orient using unique binding motifs characterized herein.

## Methods

### Structure Selection and Analysis

**Figure 1:**
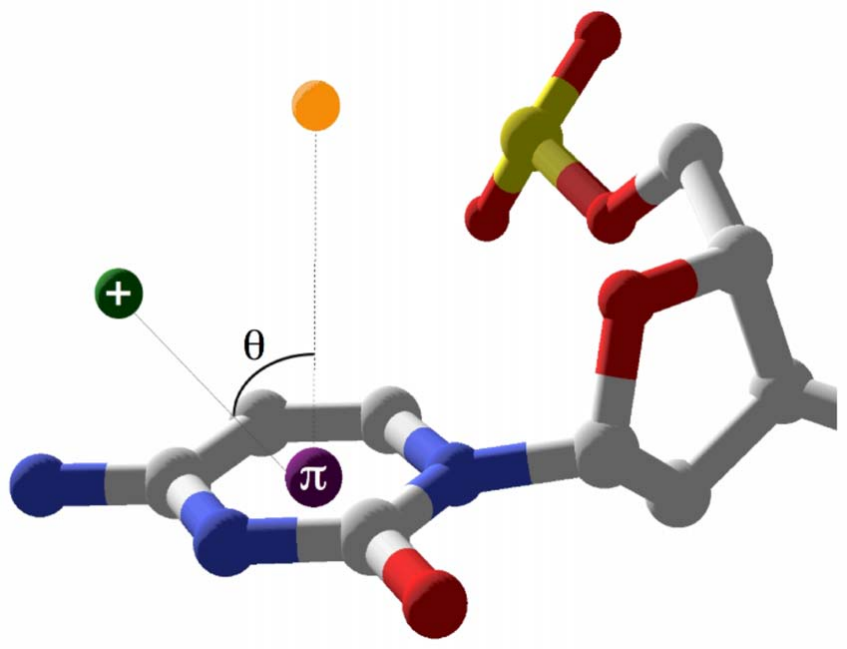
Geometric parameters defining cation-pi interactions. The distance, d, is calculated between the cation or divalent cation FSW and the centroid of the base ring represented by the purple sphere labelled p. The angle q is defined as the angle formed between the cation/FSW, the base centroid, and a point positioned normal to the plane of the base represented by the yellow sphere [3]. Parameter criteria for qualifying cation-pi interaction were d≤5.0Å and q≤50°. Terminal interaction between Tl^+^ and C1 in 1jgr is shown.

A survey of high resolution (better than 1.6 Å) B-form DNA crystal structures from the Nucleic Acid Database (NDB, [29]) was conducted. To limit artificial influences on conformation heterogeneity, structures containing base modifications, bound proteins, or ligands were discarded. Each base ring centroid coordinates were calculated and the centroid-cation/FSW distances (d) and angles from the normal to the base face (theta) were determined (Figure 1) as previously described [30].

Cation/FSWs with base centroid distances less than 5.0 Å and with theta less than 50° were tabulated and analyzed. These geometric screening criteria were more stringent than those used previously [30]. Each cation-pi interaction was then screened visually using the programs SwissPDB Viewer and Pymol. Interactions were discarded from further analysis if the base face was occluded by intervening structure/solvent atoms. Since the position of the hydrogen atoms on the water molecules is ambiguous, FSW located within hydrogen bonding distance (<3.0 Å) of two base hydrogen bond acceptors were discarded. This criterion is based on FSW-pi interaction requiring one polarized hydrogen oriented toward the base centroid, which is less likely if competing hydrogen bond acceptors are within range.

### Quantifying Base Unstacking

**Figure 2:**
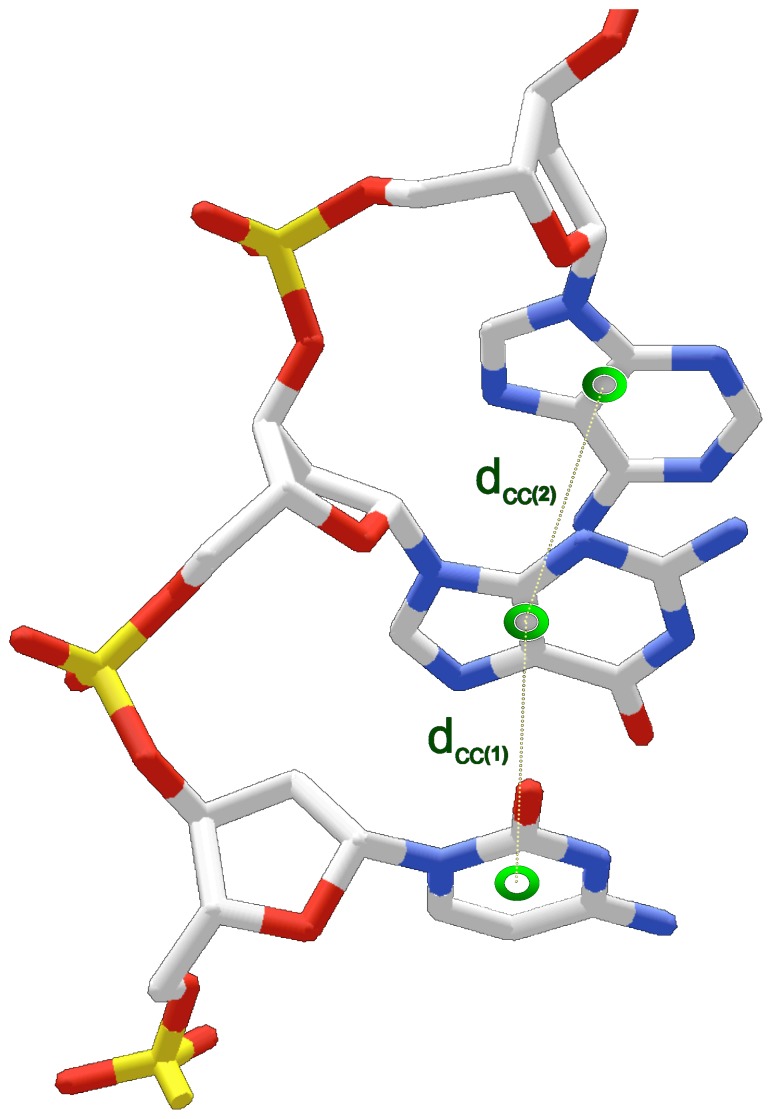
Schematic illustrating interbase center distance (d_cc_) parameters. Interbase distance between adjacent base centers (labelled 1 and 2) is calculated for each base step (d_cc_; dotted line). Calculated base centers are represented by green circles. For pyrimidines, the base center coordinates are equivalent to the ring centroid. For purines, the base center coordinates are the average of the imidazole and pyrimidine ring centroid coordinates.

Base unstacking was approximated by base-to-base distances (d_cc_) measured between base centers of adjacent nucleotides (distance  = d_cc_) (Figure 2). For purines, the base center was calculated by averaging imidazole and pyrimidine ring centroid coordinates. The base center for pyrimidines was defined by the coordinates for the ring centroid. Larger distances between base centers (d_cc_) indicate greater base unstacking. To isolate unstacking stabilized by the cation/FSW, bases involved in 1) competing interactions between ion binding in both the major and minor grooves and 2) interactions with terminal bases were omitted from d_cc_ calculations. d_cc_ values between the base involved in the cation-pi interaction and the adjacent 3’ (major groove complexes) or 5’ (minor groove complexes) base were compared with the distances (d_cc_) between adjacent, cation-pi-free (non-interacting) dinucleotides within the same structure. A base was considered non-interacting if it was free from bound ions or FSW molecules within the specified cation-pi geometry.

## Results

### Cation-pi interactions were detected in five high resolution structures

**Figure 3:**
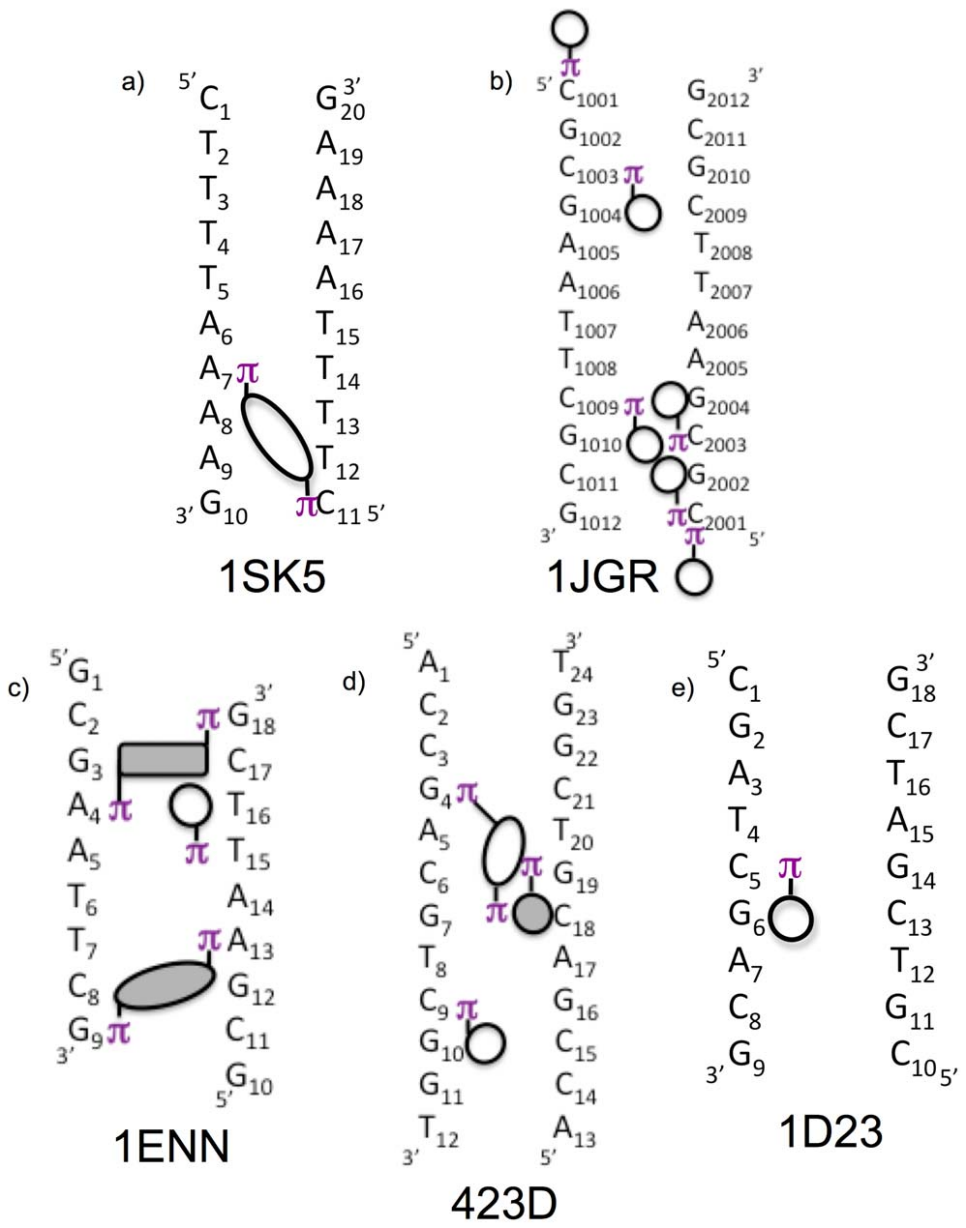
Schematic of the pattern of groove bound ions participating in cation-pi interactions. Circles and ovals/rectangles represent cations/FSWs involved in single and double cation-pi interactions respectively. Empty and shaded circles/ovals represent interactions localized to the major and minor grooves respectively. Each circle/oval is positioned adjacent to the base forming hydrogen bonds with the cation/FSW with the π next to the base involved in the cation-pi interaction. Circles/ovals here reflect only the presence of cation-pi interaction and do not distinguish between single cation/FSW interactions and those bases involved in multiple cation-pi interactions with same cations/FSWs. Rectangles reflect FSWs involved in cation-pi interactions with the bases designated with π but without hydrogen bonding to adjacent bases. In 1jgr, the circles represent Tl^+^ ions while in 1sk5 the oval represents FSWs of Ca^2+^. In the remaining structures, circles/ovals represent FSW of Mg^2+^.

Survey of the NDB produced 12 B-form DNA crystal structures containing bound ions with resolution better than 1.6 Å without chemical modifications or bound ligands. The presence of cation-pi interactions between the cations/FSWs and DNA bases was established in two subsequent screening processes as described in Materials and Methods. Initial screening of these structures for monovalent cations or FSWs against the geometric criteria (d≤5.0 Å; theta ≤50°) detected potential cation-pi interactions in 11 of these 12 structures. Subsequent application of the visual inspection criteria including proximity of cations/FSWs to hydrogen bond acceptors and unoccluded access to base centroid retained five of these structures. Within these structures, 22 cation-pi interactions are distributed among 18 distinct bases. 20 of the 22 interactions involve groove bound cations/FSW and two are formed by solvent Tl^+^ perched directly above a terminal base. The measured parameters for these cation-pi interactions are given in Table 1 and shown schematically in figure 3.

**Table 1:** Cation-pi interactions found in high resolution crystal structures.

PDB	Ion	FSW	Base	Chain	Ring	Groove	Distance (Å)	Angle (°)
1enn	MG44	48	A4	A	Pyrim	minor	5.0	48
		48	G18	B	Pyrim		5.0	50
	MG31	34	G9	A	Pyrim	minor	4.1	40
		34	G9	A	Imid		4.3	42
		32	G9	A	Pyrim		4.3	50
		32	A13	B	Pyrim		4.9	44
	19	21	T15	B	Pyrim	minor	4.8	42
1jgr	TL2101		C2003	B	Pyrim	major	4.1	27
	TL2102		C2001	B	Pyrim	major	4.4	32
	TL2110		C1009	A	Pyrim	major	4.4	37
	TL2103		C1003	A	Pyrim	major	4.2	30
	TL2111		C1001	A	Pyrim	terminal	3.6	18
	TL2112		C2001	B	Pyrim	terminal	3.6	5
423d	MG25	O3	C18	B	Pyrim	major	4.0	30
		O2	C18	B	Pyrim		4.9	36
		O1	G4	A	Imid		3.5	46
	MG26	O5	G19	B	Imid	minor	4.4	30
		O5	G19	B	Pyrim		5.0	39
	MG29	O3	C9	A	Pyrim	major	4.6	36
		O1	C9	A	Pyrim		4.5	43
1d23	MG22	423	C5	A	Pyrim	major	5.0	36
1sk5	CA306	1136	A7	A	Imid	major	4.4	27
		1135	C11	B	Pyrim		4.6	32

### A variety of cations participate in cation-pi interactions with DNA bases

Monovalent cations and first hydration shell waters of divalent cations interact with DNA pi systems (table 1; figure 3). The polarized FSWs of two divalent cations, Ca^2+^ and Mg^2+^, form cation-pi interactions in one structure (1sk5) and three structures (1enn, 423d, and 1d23) respectively. The FSWs of seven Mg^2+^ account for 14 of the 22 cation-pi interactions (63%), while the FSWs of one Ca^2+^ accounts for two.

Since the hydration shells of monovalent cations are less polarized and not well defined, Tl^+^ ions directly participate in cation-pi interactions. Na^+^ is abundant inside the cell and likely partially responsible for conformational heterogeneity imposed by electrostatic interactions between solvent ions and DNA. Because Na^+^ and water are isoelectronic and the occupancy of Na^+^ is not fixed, conclusively distinguishing Na^+^ from water directly using crystallographic methods is difficult [31]. Although Tl^+^ is not endogenous, it is commonly used as a K^+^ mimic. In addition to the unencumbered terminal cation-pi interaction described previously [4], in the structure 1jgr, six Tl^+^ ions interact via cation-pi interactions with six distinct bases.

### Sequence selective cation-pi interactions are formed within both major and minor DNA grooves

Cation-pi interactions are found in both the major and minor grooves. All five structures contain cation-pi interactions in the major groove, while two structures (1enn and 423d) also contain cation-pi interactions with Mg^2+^ FSW bound in the minor groove. Of the 22 cation-pi interactions observed, eight (36%) occur with Mg^2+^ FSW bound in the minor groove.

Cation-pi interactions form sequence selectively within the DNA grooves. All observed cation-pi interactions in these structures orient within the grooves to take advantage of the directional staircase stagger of adjacent steps inherent in the double stranded helix by forming interactions in the major groove on the 3’ side and in the minor on the 5’ side of the base (figure 3).

**Figure 4:**
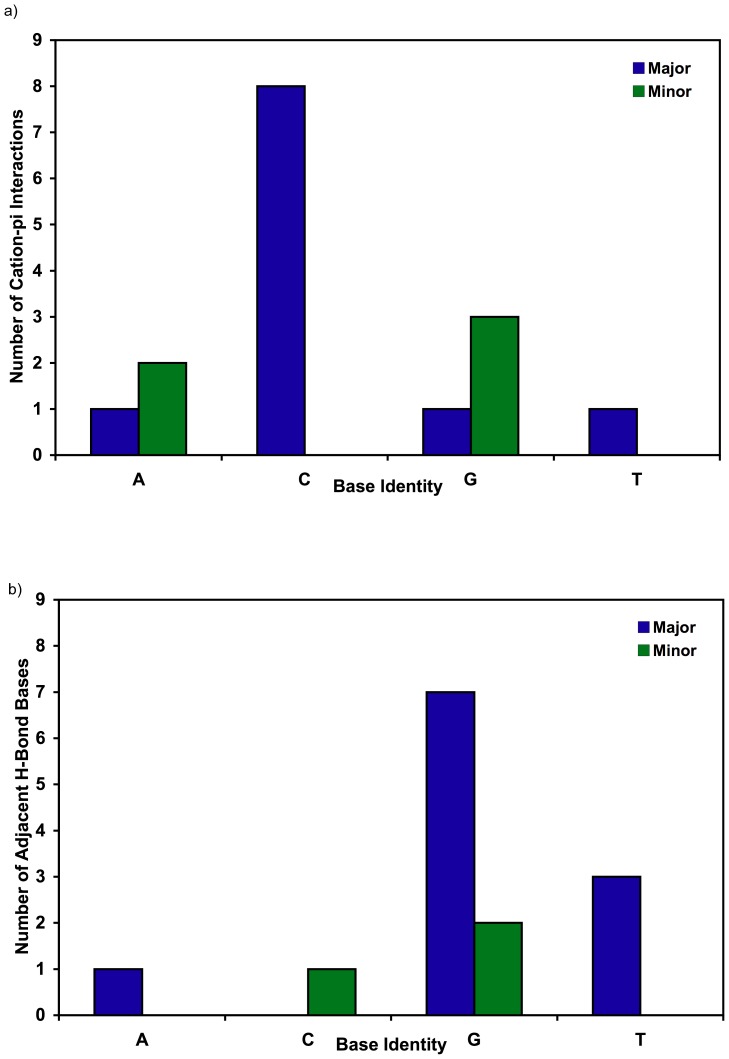
Base selectivity of cation-pi and coupled hydrogen bonding interactions. a) The number of cation-pi interactions in major (blue bar) or minor (green bar) groove is given for each of the four bases. b) The number of each base type adjacent to the base involved in cation-pi interaction(s) that also hydrogen bond or form ion/dipole interactions (1jgr) with the cation/FSW. The hydrogen bonding base is positioned 3’ for major (blue bar) and 5’ for minor groove (green bar) cation-pi interactions.

Major groove cation-pi interactions are preferentially formed with cytosine (8 of 11 bases) but single instances of interactions with A, G, and T were also observed (figure 4a). Of the two major groove purine interactions, the guanine interacts via its pyrimidine ring and the adenine via its imidazole ring. This interaction between Ca306 W1136 and A7 in 1sk5 represents the only instance of a purine imidazole ring forming a cation-pi interaction without the pyrimidine ring also residing within cation-pi distance of a cation/FSW. This exception might be due to constraints imposed by the bridging orientation assumed by the Ca306/FSW discussed further below.

The eight minor groove cation-pi interactions contained in 1enn and 423d are restricted exclusively to purines including three guanines and two adenines (figure 4a). In all cases, these interactions occur with the pyrimidine ring of the purine, while two of the guanines include additional FSW interactions with the imidazole ring.

### Hydrogen bonding with bases adjacent to cation-pi interactions extends sequence selectivity

The sequence selectivity directing cation binding is extended when adjacent bases present hydrogen bond acceptors into the groove. Bases favoring cation-pi interactions like cytosine in the major groove and the purines in the minor groove bind cations preferentially when the adjacent base is guanine in the major groove (7 of 11) and cytosine (2 of 5) or guanine (3 of 5) in the minor groove (figure 4b). This selectivity is observed whether the cation is monovalent as with Tl^+^ or divalent with a full hydration shell suggesting the selectivity is due to more than potential steric occlusion from the narrow minor grooves in AT regions.

### Cation-pi interactions with solvent cations/FSWs stabilize unstacked DNA bases

**Figure 5:**
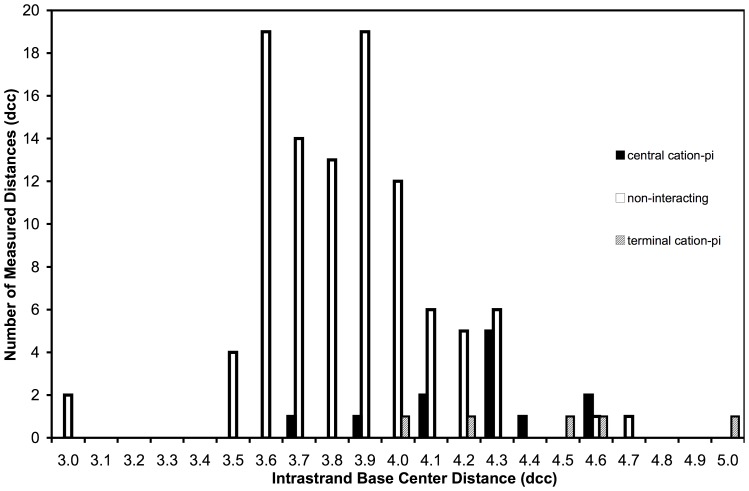
Histogram of intrastrand centroid distances. The distribution of measured d_cc_ among bases involved in cation-pi interactions with central (filled bars) or end (hatched bars) bases of DNA helices compared to bases not involved in cation-pi interactions (empty bars).

**Figure 6:**
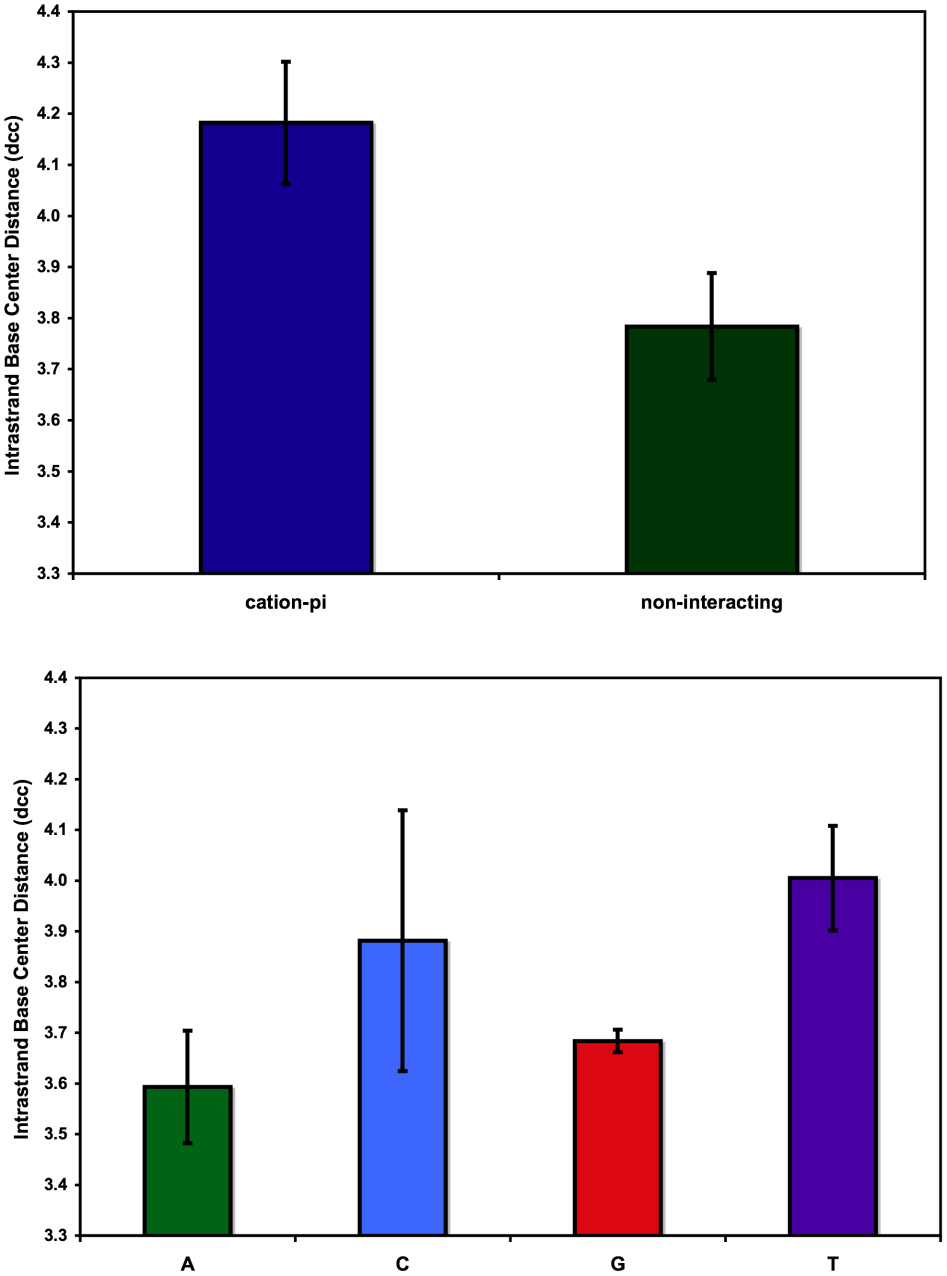
Average intrastrand centroid distances. a) Average intrastrand centroid distance (d_cc_) for bases involved in cation-pi interactions (blue) and non-interacting bases (green). b) Average d_cc_ classified by base type with adenine (green), cytosine (blue), guanine (red), and thymine (purple). Error bars reflect 95% confidence intervals.

DNA bases participating in cation-pi interactions are unstacked from the helix, exposing the pi system of the base face to the interacting cation/FSW. The distance (d_cc_) between base centers was used to estimate base unstacking as described in Methods. The frequency of individual interbase distances for bases with and without cation-pi interactions is displayed in figure 5. Bases not participating in cation-pi interactions (non-interacting, n = 102) are presented along with bases forming cation-pi interactions with either central (n = 12) or terminal bases (n = 5). A shift in the distribution toward longer d_cc_ for bases participating in cation-pi interactions is clearly visible in the histogram data. Average d_cc_ value of non-interacting bases (3.78 Å±0.10) is statistically shorter than for those bases involved in cation-pi interactions (4.18 Å±0.12) indicating that these cation-pi bases are unstacked from the helix (figure 6a) relative to those bases lacking these interactions. To confirm that variations in d_cc_ were not due to inherent mononucleotide sequence interbase distance differences, dcc data for non-interacting bases were sorted and averaged for each type of base (figure 6b). No statistically valid variation of d_cc_ was detected among the different base types suggesting no inherent mononucleotide sequence contribution. Our results suggest single base step sequence independence and do not establish a mechanistic claim concerning whether the cation-pi interactions induce unstacking or stabilize inherently unstacked conformations dictated by specific consecutive bases. Further investigations into the mechanism of the observed unstacking, including its dependence on interbase pair parameters will be necessary.

### Monovalent Tl^+^ ions perched on terminal cytosines display optimal cation-pi geometry

**Figure 7:**
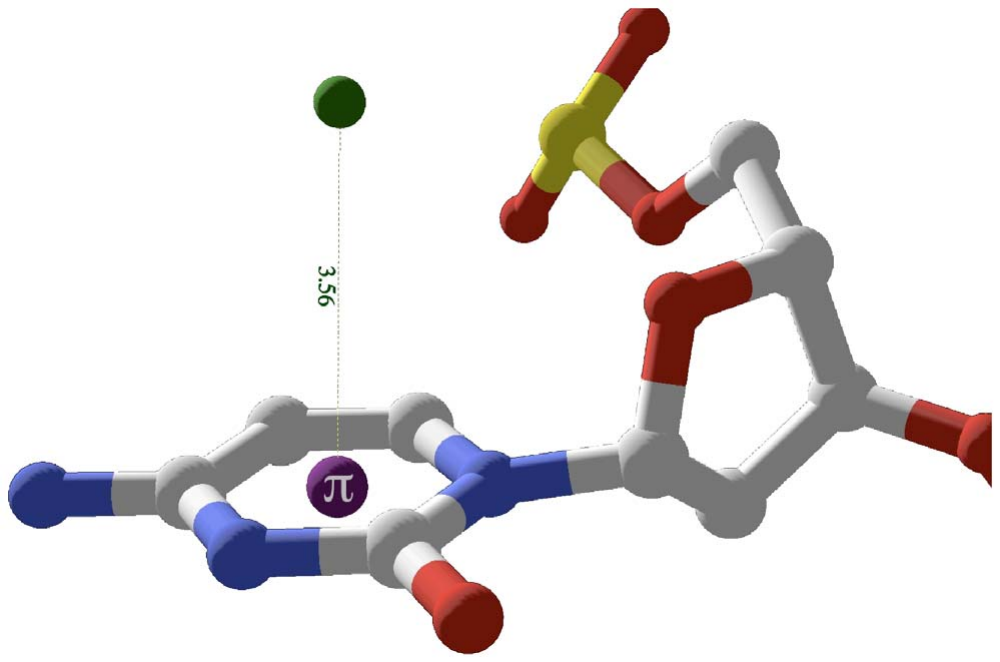
Terminal Tl^+^ cation-pi interactions in 1jgr. TL2112 is perched directly above the centroid of the terminal base C2001. The interaction is optimal since the ion position is sterically unencumbered and in less competition with stacking interactions from flanking bases. For the interaction shown, TL2112 d = 3.56 Å, ƒ = 4.7°.

Monovalent Tl^+^ ions perched over terminal cytosines display unencumbered cation-pi geometry. Although these terminal ions compete with lattice interactions [4], the geometry displayed is optimal with the average of the two positions having d_ave_ = 3.56 Å and theta_ave_ = 11.5°. Figure 7 displays TL2112 perched above terminal base C2001 with d = 3.56 Å and theta = 4.7° from 1jgr.

### Monovalent cations and FSWs in the major groove primarily occupy intrastrand CG tridentate binding pocket

**Figure 8:**
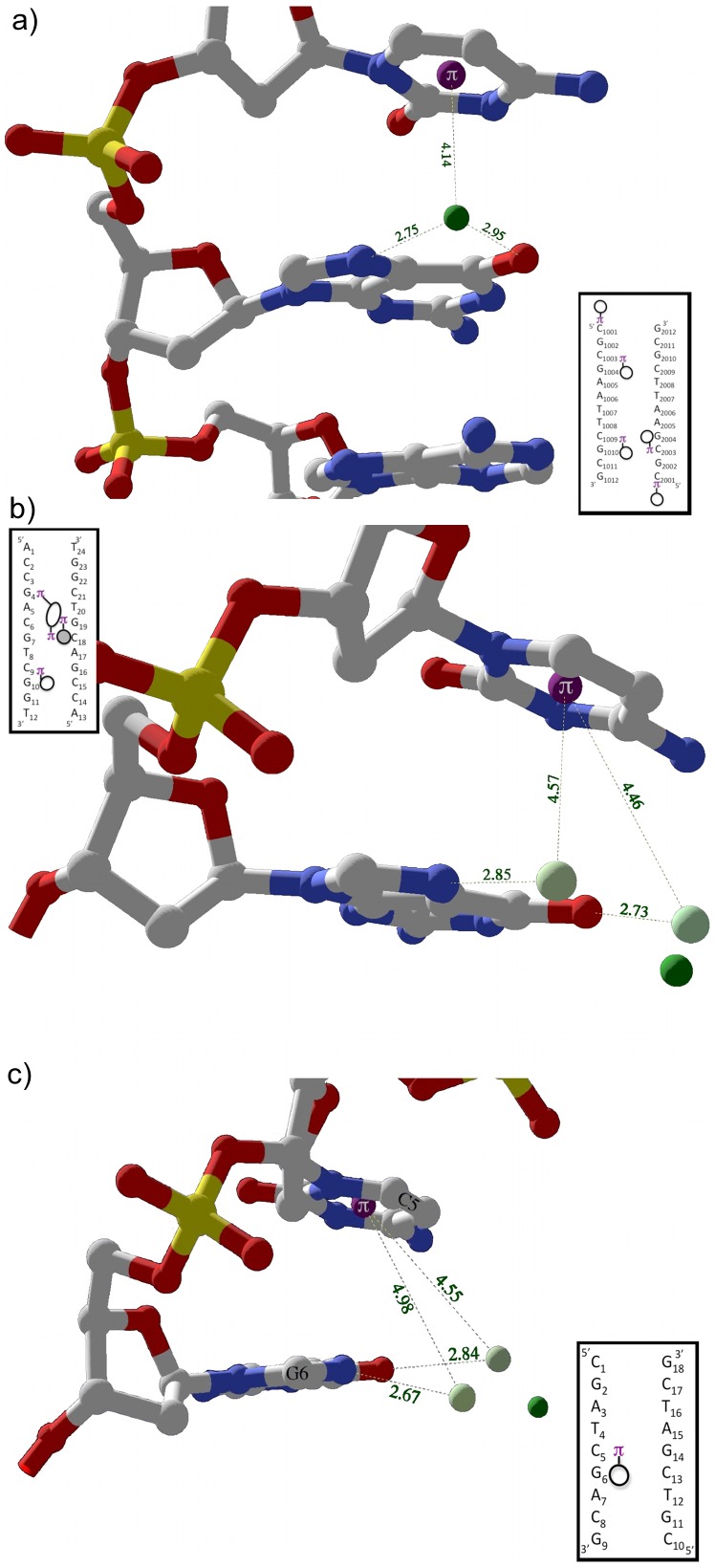
Intrastrand tri-dentate cation/FSW orientations in the major groove. a) One of three examples in 1jgr between TL2101 and a CG step. The Tl^+^ ion forms an ion-dipole interaction with O6 and N7 of G2004 while simultaneously interacting with the pi system of C2003. Inset is interaction schematic for 1jgr. b) Example in 423d between Mg29 FSWs and a CG step. Two Mg29 FSW, W520 and W518 hydrogen bond with N7 and O6 of G10 respectively while both form cation-pi interactions with the adjacent base C9. c) Side view of two FSW of Mg22, W143 (proximal) and W147 (distal) interaction, through hydrogen bonds with N7 and O6 of G6 respectively and cation-pi with neighboring C5 at the distances shown. FSW (light green); ions (dark green); base ring centroid (purple).

All three monovalent Tl^+^ and 2 of 3 divalent cation FSW intrastrand cation-pi interactions in the major groove selectively bind to CG steps. Because monovalent cations have small radii and hydration shell waters that are less tightly bound, the ions bind snugly in an intrastrand pocket that offers stabilizing tridentate electrostatic interactions including cation-pi interaction with the 5’ cytosine and simultaneous ion-dipole interactions with O6 and N7 of the 3’ guanine. Figure 8a shows an example of this tridentate interaction involving TL2010 and C2003 with ion-dipole interactions with O6 (2.95 Å) and N7 (2.75 Å) of G2004 and cation-pi interaction with 5’ C2003 (d = 4.14 Å; theta = 27°).

Two intrastrand tridentate CG binding motifs were observed for major groove MgFSWs with hydrogen bonds between guanine N7/O6 and two cis oriented FSWs replacing Tl-G ion-dipole interactions. In both cases, two cis-FSW form cation-pi interactions with 5’ cytosine. In structure 423d, two Mg29 FSWs, W03 and W01, hydrogen bond with N7 (2.85 Å) and O6 (2.73 Å) of G10 respectively. Both W03 (d = 4.57 Å; theta = 36°) and W01 (d = 4.46 Å; theta = 43°) form cation-pi interactions with the adjacent 5’ base C9. (figure 8b).

In structure 1d23, two Mg22 FSWs, W143 and W147, hydrogen bond with N7 (2.67 Å) and O6 (2.84 Å) of G6 respectively (figure 8c). Both W143 (d = 4.48 Å; theta = 36) and W147 (d = 4.55 Å; theta = 53°) fall within the distance criteria, while W147’s angle is slightly greater than the cutoff of 50°. Though technically not a cation-pi interaction according to our conservative criteria and therefore not included in table 1 or any of the statistics reported, W147 is included here because 1) this water is positioned closer to the ring centroid of C5 than is W143, whose geometry falls within both d and theta limits, and 2) binding pattern characterization often depends on qualitative considerations in addition to geometric criteria applied.

### Other intrastrand MgFSW orientations include major groove bidentate and minor groove inverse tridentate examples

**Figure 9:**
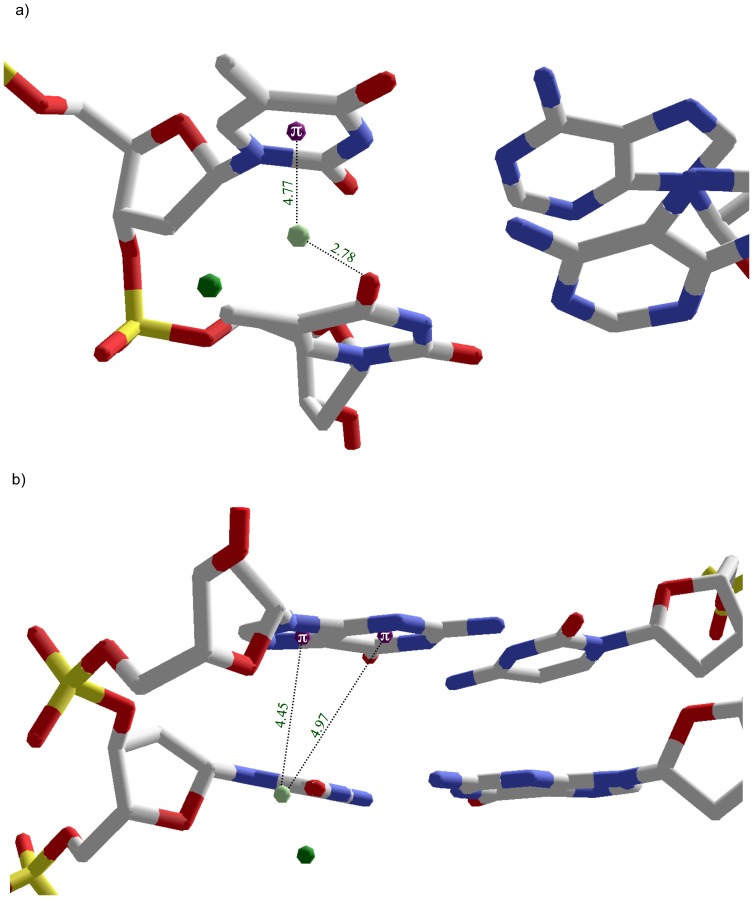
Intrastrand bidentate and reverse tridentate cation/FSW orientations. a) Bidentate orientation in 1enn between Mg19 FSW and TT step in which W21 simultaneously participates in a cation-pi interaction with T15 while hydrogen bonding with O4 of T16. b) Reverse tridentate orientation in the minor groove of 423d between Mg26 FSW and GC step in which W501 hydrogen bonds with O2 of C18 and forms cation-pi interactions with both the imidazole and pyrimidine ring of G19. FSW (light green); Mg^2+^ (dark green); base ring centroid (purple).

One major groove bidentate intrastrand binding motif is observed at a TT step in 1enn. Mg19 FSW W21 simultaneously forms a cation-pi interaction with T15 (d = 4.8 Å; theta = 42°) and hydrogen bonds with O4 of T16 (2.78 Å). The combination of T15’s base pi system with respect to T16’s O4 positions Mg19 almost in the center of the groove floor rather than the distinct sideline orientation provided by the tridentate CG binding pocket (figure 9a).

The only isolated minor groove intrastrand cation-pi binding pocket observed involves an inverse tridentate orientation occurring at a GC step. This inverse tridentate orientation consists of a single FSW simultaneously hydrogen bonding with the 3’ cytosine and within cation-pi geometry of both rings of the 5’ guanine. Specifically, in structure 423d a single FSW, W05, of Mg26 hydrogen bonds with the O2 of C18 (2.83 Å). This same water satisfies the cation-pi criteria for both the imidazole (d = 4.4 Å; theta = 30°) and pyrimidine (d = 5.0 Å; theta = 35°) rings of G19 (figure 9b). Assuming one of the hydrogens of W05 is hydrogen bonding with C18, the remaining hydrogen can favorably interact with either G19 ring but not both simultaneously unless a position between two ring centroids allows favorable interaction with the delocalized pi system of the purine.

### Divalent cations form interstrand bridges in DNA grooves via multiple bi- and tridentate arrangements

**Figure 10:**
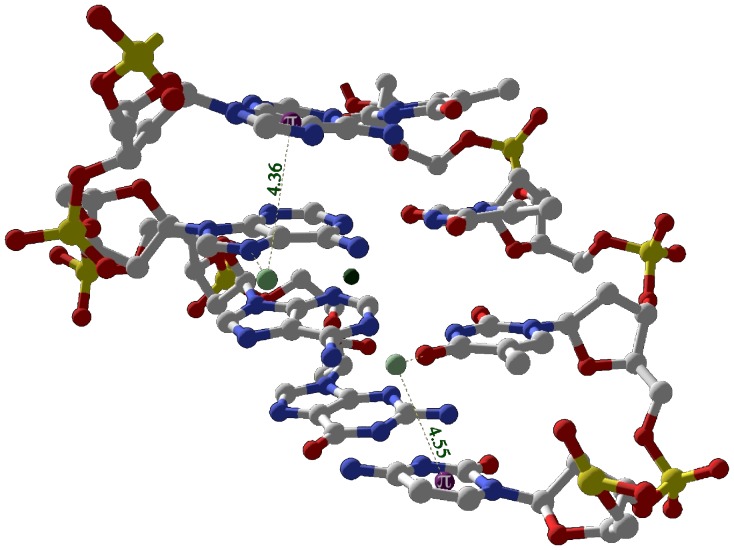
Interstrand bridging via bidentate orientations in the major groove of 1sk5. Two Ca306 FSWs, W1135 and W1136, form bidentate interactions with bases on opposing strands. W1135 simultaneously forms a cation-pi interaction with C11 and a hydrogen bond with O4 of adjacent T12. W1136 forms a cation-pi interaction with the imidazole ring of A7 and a hydrogen bond with N7 of A8 on the opposing strand. FSW (light green); Mg^2+^ (dark green); base ring centroid (purple).

In the major groove, bridging interstrand interactions involving both calcium and magnesium ions are built by FSWs forming either bi- or tridentate cation-pi and/or H-bond interactions with bases on opposing DNA strands. Two Ca306 FSWs in structure 1sk5, W1135 and W1136, form bidentate interactions with bases on different strands (figure 10). W1135 forms a cation-pi interaction (d = 4.55 Å; theta = 32°) with C11 while hydrogen bonding with O4 (2.71 Å) of adjacent T12. W1136 forms a cation-pi interaction (d = 4.36 Å; theta = 28°) with imidazole ring of A7 and hydrogen bonds with N7 (d = 2.78 Å) of A8 on the opposite strand.

In structure 423d, the major groove bridge is formed by Mg25 FSW interstrand bi- and tridentate interactions with bases on opposite strands. The tridentate interaction formed by two FSW, W02 and W03, which hydrogen bond to the N7 (2.84 Å) and O6 (2.80 Å) of G19 respectively and simultaneously form cation-pi interactions with C18 (see table 1). The bridging bidentate interaction involves W01 of the same Mg25 forming a cation-pi interaction (d = 3.46 Å; theta = 46°) with the imidazole ring of G4 on the opposite strand while hydrogen bonding with O4 (2.68 Å) of T20.

In the minor groove, bridging interstrand interactions are assembled from a combination including bidentate and inverse tridentate interactions. Two sets of bridging minor groove interactions are formed in 1enn. In both cases, the narrow minor groove allows a FSW to simultaneously participate in cation-pi interactions with an adenine and a guanine on opposing strands. Participation in dual cation-pi interactions should preclude simultaneous hydrogen bonding. As expected, these FSWs are not within optimal orientation or interaction distance of any hydrogen bond acceptors. Because the cation-pi interactions involve the minor groove side of the pyrimidine ring of the purines, the N3 position is positioned toward the FSWs. However, in both cases the distance is longer than those observed for optimal hydrogen bonding and the waters are oriented above or below the pyrimidine ring, disfavoring interaction with the planar N3 lone pair.

**Figure 11:**
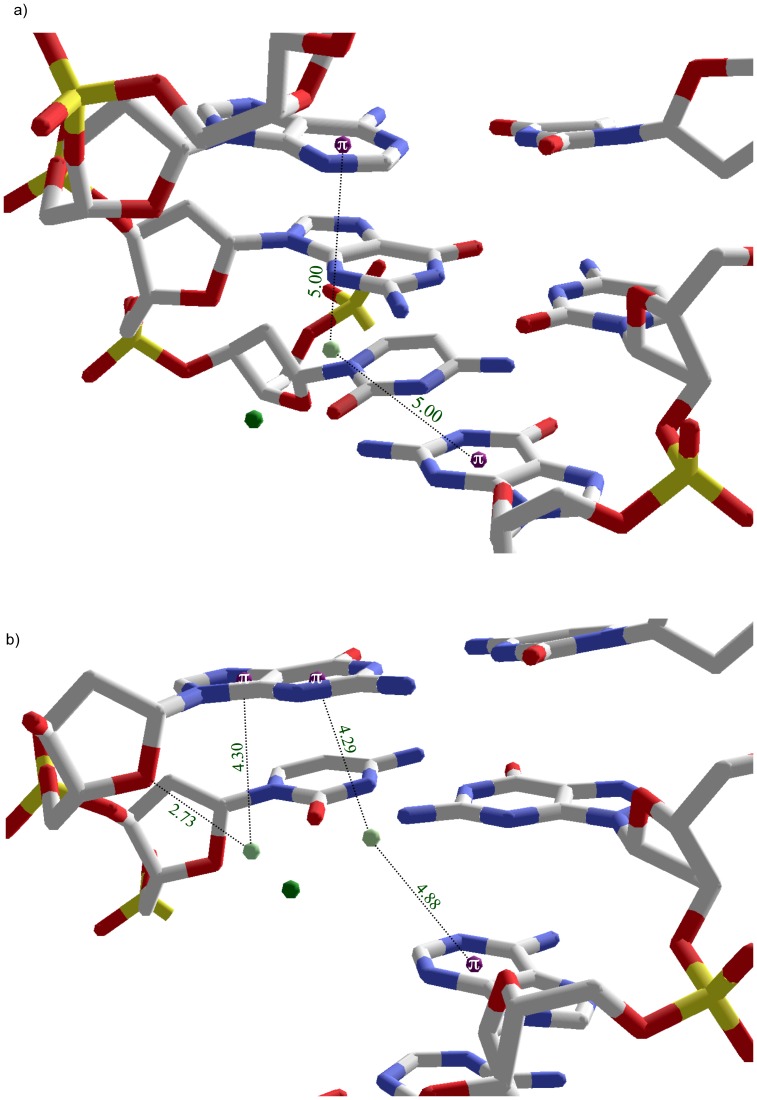
Interstrand bridging via combinations of reverse tridentate and dual cation-pi interactions in minor groove of 1enn. a) Mg48 FSW W32 maintains a dual cation-pi orientation through interactions with the pyrimidine rings of A4 and G18 on opposing strands. b) Interstrand bridging of G9 and A13 by Mg31 FSWs. W32 forms dual cation-pi interactions with the pyrimidine rings of A13 and G9. W34 demonstrates an inverse tridentate interaction by forming cation-pi interactions with both the imidazole and pyrimidine rings of G9 and hydrogen bonding with O4’ of G9. Interacting FSW (light green); Mg^2+^ (dark green); base ring centroids (purple).

Mg48 FSW W32 forms an example of this dual cation-pi orientation including interactions with the pyrimidine rings of A4 (d = 5.0 Å; theta = 48°) and G18 (d = 5.0; theta = 50°) (figure 11a). Mg31 has two FSWs, W32 and W34, that form the interstrand bridge. W32 forms dual cation-pi interactions with the pyrimidine rings of A13 (d = 4.9 Å; theta = 44°) and G9 (d = 4.3 Å; theta = 50°). W34 forms an inverse tridentate interaction including cation-pi interactions with both the imidazole and pyrimidine rings of G9 (table 1) while hydrogen bonding with O4’ of G9 (figure 11b).

## Discussion

A variety of cations, either directly or via their FSWs, bind sequence selectively in both DNA grooves via the combination of cation-pi and ion-dipole/H-bonding interactions. Our data allow us to begin to decipher the solvent cation pattern oriented on the groove floor by a combination of sequence selective cation-pi and electrostatic interactions. Here are some conclusions we can draw from the data presented herein.

### Cations/FSWs binding motifs include a combination of cation-pi and ion-dipole/H-bonding interactions

Cations bind within the DNA grooves with sequence selectivity provided by directional orientation of consecutive bases offering favorable cation-pi interactions juxtaposed with a base presenting hydrogen bond acceptors, through which the bound cation or its FSWs can electrostatically interact, into the groove floor. Such binding pockets provide simultaneous stabilizing interactions that direct the cations into position.

### Cation-pi interactions form preferentially at CG steps in the major groove and with purines in the minor groove

Ten of the eleven major groove bound cations include cytosine as a cation-pi participant, with eight of the 11 including cytosine exclusively (figure 4). Two of the exceptions to cytosine include cation-pi interactions with purine imidazole rings (G4 in 423d and A7 in 1sk5) whose interacting cation’s FSW also have a cytosine cation-pi interaction as their interstrand bridging partner (figure 3). In most cases (7 of 8 occurrences) the 3’ base adjacent to a cytosine participating in cation-pi interaction is a guanine. The hydrogen bond acceptors O6 and N7 on the major groove edge of the adjacent guanine provide additional interaction sites for cations/FSWs.

Cation-pi interactions form exclusively with purines in the minor groove. Bridging cation FSWs form cation-pi interactions with an adenine and a guanine. One minor groove intrastrand cation-pi interaction is with G19 in 423d. In all but one case, the minor groove cation-pi interactions are exclusively with the pyrimidine ring of a purine. It is interesting to note that the FSW forming the cation-pi interaction with the one imidazole ring (G9 in 1enn) also forms a more optimal cation-pi interaction with the pyrimidine ring of the same base in a reverse tridentate orientation. Hydrogen bonding between the cations/FSWs and base edges appears to be a less important stabilizing factor for ions bound in the minor groove. Perhaps this is due to the close proximity of the negatively charged phosphate backbone of the narrow groove providing electrostatic stabilization.

### Cation-pi interactions with cations/FSWs stabilize unstacked conformations of DNA bases

DNA bases involved in cation-pi interactions tend to be pulled away from the helical stack toward the cation/FSW. Intrastrand base center distances were used to estimate base unstacking and interacting bases have longer intrastrand distances on average. The previous observation that Mg^2+^ FSW cation-pi interactions appear to stabilize unstacked conformation of cytosines in a high resolution crystal structure of the Dickerson dodecamer [30] is supported in our survey of cation-pi interactions between solvent cations/FSWs and DNA bases.

### Major groove floor arrangement is built from distinct cation-pi/electrostatic binding motifs established by DNA sequence

Intrastrand tridentate and inverse tridentate motifs in the major groove position the cation to the groove floor sideline. Cation-pi interactions in the major groove preferentially form tridentate interactions especially at CG steps. One instance of an inverse tridentate interaction was also observed in the major groove. In both cases, dual interactions with either the O6 and N7 atoms (tridentate) or the pyrimidine and imidazole rings (inverse tridentate), orient the cation to one side of the groove floor, allowing either the monovalent ion or divalent cation FSWs to interact simultaneously via cation-pi and ion-dipole/hydrogen bond interactions. Though beyond the scope of this work, the sum of the three binding energies is likely substantial enough to tether the ion to its position in the groove.

Intrastrand bidentate motifs in the major groove seem to position the cation towards the center of the groove floor. Though limited by a single instance in the present study, a single bidentate interaction observed in the major groove occurs in 1enn at a TT step. A single FSW forms a cation-pi interaction with the 5’ T5 while hydrogen bonding with the O4 of the T4. The orientation of this hydrogen bond acceptor shifts the ion position toward the center of the groove floor rather than the sideline as observed with intrastrand tridentate interactions.

Interstrand bridging motifs are centered on the major groove floor and seem to stitch the strands together through simultaneous FSW cation-pi/H-bonding interactions with both strands. Support for the major groove cation-pi preference for cytosine continues, as cytosine is one of the interstrand partners in both major groove-bridging orientations observed. This interesting observation suggests that the preferential cytosine cation-pi interaction serves as an anchoring interaction for the interstrand bridge. These cases present a limited but intriguing pattern suggesting sequence selectivity with bridges forming in the major groove between a cytosine and a purine’s imidazole ring in the n-3 position on the opposing strand.

The minor groove likely restricts cation-pi interactions involving divalent cations to the relatively wider CG regions while the inherent narrowness of the groove promotes bridging by FSWs. While narrow minor groove floor of AT regions have been shown to bind monovalent cations, which serve as a foundation for interesting water assemblies that protrude from the minor groove floor [32], [33] like a mohawk, the binding of divalent cations with their hydration shell would likely be sterically prohibited in these regions. Indeed, all minor groove cation/FSW cation pi interactions observed in this study include interactions with guanines. Interestingly, the two bridging interstrand interactions occur at AT-CG junctions and include cation-pi interactions with both purines. These bridging interactions seem to suggest some sequence selectivity by forming between adenine and guanine with one purine positioned in the n+2 position on the opposing strand with respect to the other. The narrow groove allows a single FSW to simultaneously form dual cation-pi interactions with the pyrimidine rings of the purines. However, all of these minor groove bridging motif observations were limited to two examples within a single structure, so this sequence selectivity remains only speculative.

### Speculative role of solvent cation binding motifs in protein-DNA complex formation

Cation-pi and electrostatic interactions between solvent cations and DNA bases are similar and possibly mimic the interactions observed in protein-DNA complexes. Pyrimidine-guanine base steps (YG) are sites of increased helical flexibility and often sites of helical bending, [34], [35] which have been shown to be at least partially dependent on cation binding [36], [37], [38], [39]. Lamoureux, et al reported that positively charged protein amino acid side chains, arginine and to a lesser degree histidine, form cation-pi interactions at YG steps in their DNA target sequences and that these interactions unstack pyrimidines from the helical stack [40]. Rooman, et al detected the prevalence of stair motifs in protein-DNA complexes in which primarily arginine side chains form simultaneous cation-pi and hydrogen bonding interactions with adjacent bases [41], [42]. Recently, Zou, et al used elegant computational analysis to detect stair motifs at methylated CG steps in protein-DNA complexes involved in DNA repair [21].

These stair motifs in protein-DNA complexes are strikingly similar to the tridentate binding motif described herein. For monovalent cations, the positively charged Tl^+^ ions are positioned to directly interact with the hydrogen bond acceptors O6 and N7 of guanine while interacting with the aromatic pi electrons of the 5’ cytosine. For divalent cations, two polarized divalent cation FSWs share the role of the planar arginine guanidinium side chain, which contains a delocalized positive charge.

The surprising similarity of these patterns in protein-DNA complexes to our findings in DNA solvent cation complexes supports our conclusions and offers an intriguing possibility that the pattern of cations bound in the DNA grooves serves a couple of related roles including mimicry and place holding that could assist the protein in docking to its target sequence. First, this patchwork pattern of cations in the groove could mimic the binding patterns that will be assumed by cationic protein side chains thereby helping to communicate the details of the underlying DNA target sequences to the searching protein. Second, these mimicking solvent cations could also serve as energetic placeholders, paying part of the energetic price for the DNA conformational distortion that would need to be induced by the searching protein when binding to its DNA target.
